# An Unusual Case of Retained Foreign Body in the Vagina With Vaginal Fibrosis

**DOI:** 10.7759/cureus.20956

**Published:** 2022-01-05

**Authors:** Subrat Panda, Ananya Das, Rituparna Das, Nalini Sharma, Vinayak Jante

**Affiliations:** 1 Obstetrics and Gynecology, North Eastern Indira Gandhi Regional Institute of Health and Medical Sciences (NEIGRIHMS), Shillong, IND

**Keywords:** fibrosis, vaginal discharge, foreign body, vaginal fibrosis, vaginal foreign body

## Abstract

Vaginal foreign bodies can cause long haul, foul-smelling vaginal discharge and vaginal bleeding and are typically found in female youngsters while looking into vaginitis and urinary tract diseases. There are many causes for vaginal discharges. Among them, vaginal foreign bodies are uncommon but not a very rare presentation. We had a case of a 49-year-old female, a widow, with para 2 living 2 and a history of menopause since three years; she was referred from a district hospital with a diagnosis of carcinoma of the cervix and was later found to have a foreign body, which was removed surgically through the vagina. A foreign body in the vagina is usually seen in children than in adults. Foreign bodies are inserted vaginally for treatment purposes, contraception, induced abortion, and sexual stimulation in adults. Here, we report a case of retained vaginal foreign body with vaginal fibrosis.

## Introduction

Vaginal foreign bodies are typically found in female youngsters while looking into vaginitis and urinary tract diseases [1]. A foreign body in the vagina is usually seen in children than in adults. Foreign bodies are inserted vaginally for treatment purposes, contraception, induced abortion, and sexual stimulation in adults. Almost all the foreign bodies found inside the vaginal canal would not have been inserted without the knowledge of that woman. They can be inserted for iatrogenic purposes and sexual gratification or by a third person in an event of sexual assault. There are many causes for vaginal discharges. Among them, vaginal foreign bodies are uncommon but not a very rare presentation [2,3].

## Case presentation

We had a case of a 49-year-old female, a widow, with para 2 living 2 and a history of menopause since three years; she was referred from a district hospital with the diagnosis of carcinoma of the cervix stage IIB. Her complaint was of a dirty foul-smelling discharge for one year and intermittent bleeding per vaginum. She went to the local PHC and from there was referred to a nearby district headquarter hospital. In that district headquarter hospital, she was diagnosed with a case of carcinoma of the cervix with an MRI report supporting the diagnosis without the biopsy report. From the district headquarter hospital, she was referred to our hospital for further management. She attended our outpatient department (OPD). Her vital signs were normal. The abdomen was soft. On per speculum examination, only a little portion of the external os was seen, which cannot be held with any instrument. No fornix was visible. The external os visible was unhealthy, and a foul-smelling discharge was present. Both side fornices were not visible. There was a small opening in the right side of the vagina, and only a little finger can be accommodated into it. Therefore, the little finger was introduced through the opening, and there was a small space where a plastic ringlike structure was felt. A liquid Pap smear was taken. On per vaginal examination, we could not feel the uterus and cervix. On per rectal assessment, rectal mucosa was free. The cervix could not feel properly. Fibrosis was felt on both sides. Antibiotic was started. The Pap smear was negative for intraepithelial lesion or malignancy (NILM) and diffuse inflammatory smear. On ultrasonography, the finding was a bulky cervix, which correlates clinically. Therefore, the decision of examination under anesthesia was taken. She did not give any history of sexual activity. She avoided more focused questions possibly due to embarrassment by claiming that she has difficulty recalling past events, for which we obliged and omitted detailed history thereafter.

**Figure 1:**
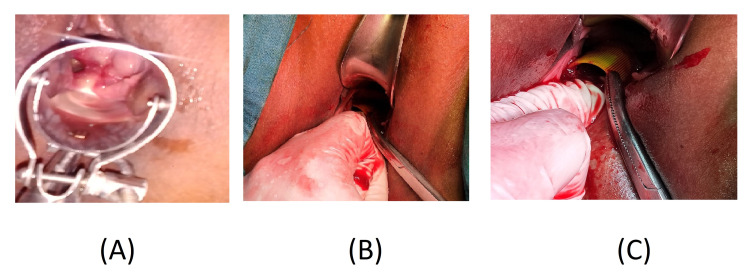
(A) Per speculum examination in OPD. (B) After removing vaginal septum-like fibrosis, the foreign body is seen. (C) Removal of the plastic cap (foreign body).

On examination under anesthesia, the small opening in the right side of the vagina was extended, and a plastic bottle cap was found; it was in the right fornix (Figure 1). The surrounding was a fibrosed area, and the right and left side fornices were cleared. The cervix was cleared by pushing the bladder, and the external os was cleared. The uterine sound was introduced to the uterine cavity by ultrasound guidance. She tolerated all the procedures, and the postoperative period was uneventful.

## Discussion

In our 22 years of experience in medical college, this was our second encounter with a case of vaginal foreign body. The first case was of an incarcerated wooden apple inserted for prolapsed uterus in a 75-year-old female, and the present study was the second case. In the first case, fibrosis was not present, but in the present case, fibrosis looked like a complete vaginal septum within the middle tip of the external os. Foreign bodies in the vagina can cause different complications, such as injury and perforate vagina, and may go into the bladder, causing peritonitis and pelvic and vaginal adhesion, and develop into fistulas in the bowels, bladder, uterus, and vagina. Foreign bodies might cause vaginitis, causing vaginal ulceration, and involve adjoining constructions such as the bladder and rectum, causing urinary and fecal incontinence [4-6]. Foreign body in situ may cause chronic inflammation and fibrosis, which leads to vaginal stenosis and complete obstruction eventually [7]. Ascending infections can give rise to endometritis, salpingitis, and peritonitis. The diagnosis of vaginal foreign bodies is based on history and thorough examination, especially speculum examination and bimanual digital vaginal examination. Sometimes, imaging studies can be useful for the identification of foreign bodies, especially when there is an unexplained purulent or bloody vaginal discharge [8]. MRI is the best accessible imaging method to assess vaginal foreign bodies [9].

Vaginal foreign bodies account for approximately 4% of gynecological complaints in prepubertal girls [10]. The most common foreign body found in prepubertal girls is toilet tissue paper. Sometimes, other objects have also been reported, such as small toys, safety pins, and other small objects [11]. Most of the time, they are unable to provide a clear history, while a few will come up with a history of placing an object in their vagina. No matter what the history is, a thoroughly detailed history study has to be taken, and an examination should be done because there is a high chance of sexual abuse, and it should be excluded all the time.

The ideal management of vaginal foreign bodies is the removal from the vaginal canal. If it is uncomplicated, it can be removed easily without anesthesia. Certain objects such as sharp nails and hazardous objects may need anesthesia to remove them carefully. In some instances, instruments such as obstetrics forceps and vacuum devices may be required for the removal. Rarely, laparotomy is performed in complicated cases. Vaginoscopy with anesthesia may be required for children.

## Conclusions

Vaginal foreign bodies are present in female patients of all ages. When evaluating a patient with a suspected vaginal foreign body, history should focus on the details surrounding the initial event; this includes the timing, suspected object, and symptoms of the abdomen, pelvis, and genitalia. History-taking is imperative in all patient populations. It is essential to consider sexual abuse as a cause for foreign bodies, especially in the pediatric population. In women of all ages with vaginal discharge, retention of a foreign body may not be a common diagnosis but should not be a rare diagnosis.
